# The chick embryo chorioallantoic membrane as an experimental model to study lung cancer

**DOI:** 10.3389/fonc.2026.1758487

**Published:** 2026-01-29

**Authors:** Domenico Ribatti

**Affiliations:** Department of Translational Biomedicine and Neuroscience, University of Bari Medical School, Bari, Italy

**Keywords:** angiogenesis, anti-angiogenesis, chorioallantoic membrane, *in ovo*, lung cancer, tumor biology

## Abstract

The chick embryo chorioallantoic membrane (CAM) has long been a favored system for the study of tumor growth because a chick’s immunocompetent system is not fully developed, and the conditions for rejection have not yet been established. Grafting tumors onto the CAM allows us to study the morphological aspects of the interactions of the tumors with the blood vessels of the host and to examine the identity of the vessels that supply the grafts. This article analyzes the literature data concerning the use of the CAM model to study lung cancer and the effects of anti-angiogenic molecules on the growth of this tumor due to the lack of literature data summarizing this topic.

## Introduction

The chick embryo chorioallantoic membrane (CAM) is formed at embryonic days (EDs) 3–4 by the fusion of the chorion and the allantois, and it consists of three layers: ectoderm (from the chorion), mesoderm, and endoderm (from the allantois) (1). In this double layer, an extremely rich vascular network develops, which is connected to embryonic circulation by two allantoic arteries and one allantoic vein. By ED 16, the CAM becomes closely pressed against the shell membranes, which enables it to act as a gas-exchange organ receiving oxygen and eliminating carbon dioxide through the pores in the shell (1). Immature blood vessels scattered in the mesoderm grow very rapidly until ED 8 and give rise to a capillary plexus, which comes to be intimately associated with the overlying chorionic epithelial cells. At ED 14, the capillary plexus is located at the surface of the ectoderm adjacent to the shell membrane. Endothelial cell mitotic index declines rapidly, and the vascular system attains its final arrangement on ED 18, just before hatching. On EDs 10–12, the mesodermal vessels are now distinct arterioles and venules accompanied by a pair of interconnected lymphatics (2). Veins are also associated with lymphatics, and larger veins are surrounded by a lymphatic plexus (3).

## The CAM assay in the study of tumor biology

The CAM has long been a favored system for the study of tumor growth because a chick’s immunocompetent system is not fully developed, and the conditions for rejection have not yet been established (4). In 1911, Rous demonstrated the growth of the Rous sarcoma grafted onto the CAM (5). In 1912, Murphy reported that mouse and rat tumors implanted onto the CAM could be maintained by a continuous passage from egg to egg (6). The first evidence of tumor-induced angiogenesis *in vivo* was obtained using the CAM assay, dated 1913 (7). Starting from these observations, the CAM has been established as an experimental system for research in tumor biology. The behavior of chicken, mouse, and human tumor cells and tissues implanted on the CAM surface was compared and evaluated for their growth, histological features, viability after re-transplantation in their original host, and the effects on the chick embryo (8). The CAM has been used as a test system for tumor chemosensitivity (9) and for the study of tumor invasion and metastasis and of neovascularization of heterologous normal and neoplastic implants (10).

**Figure 1:**
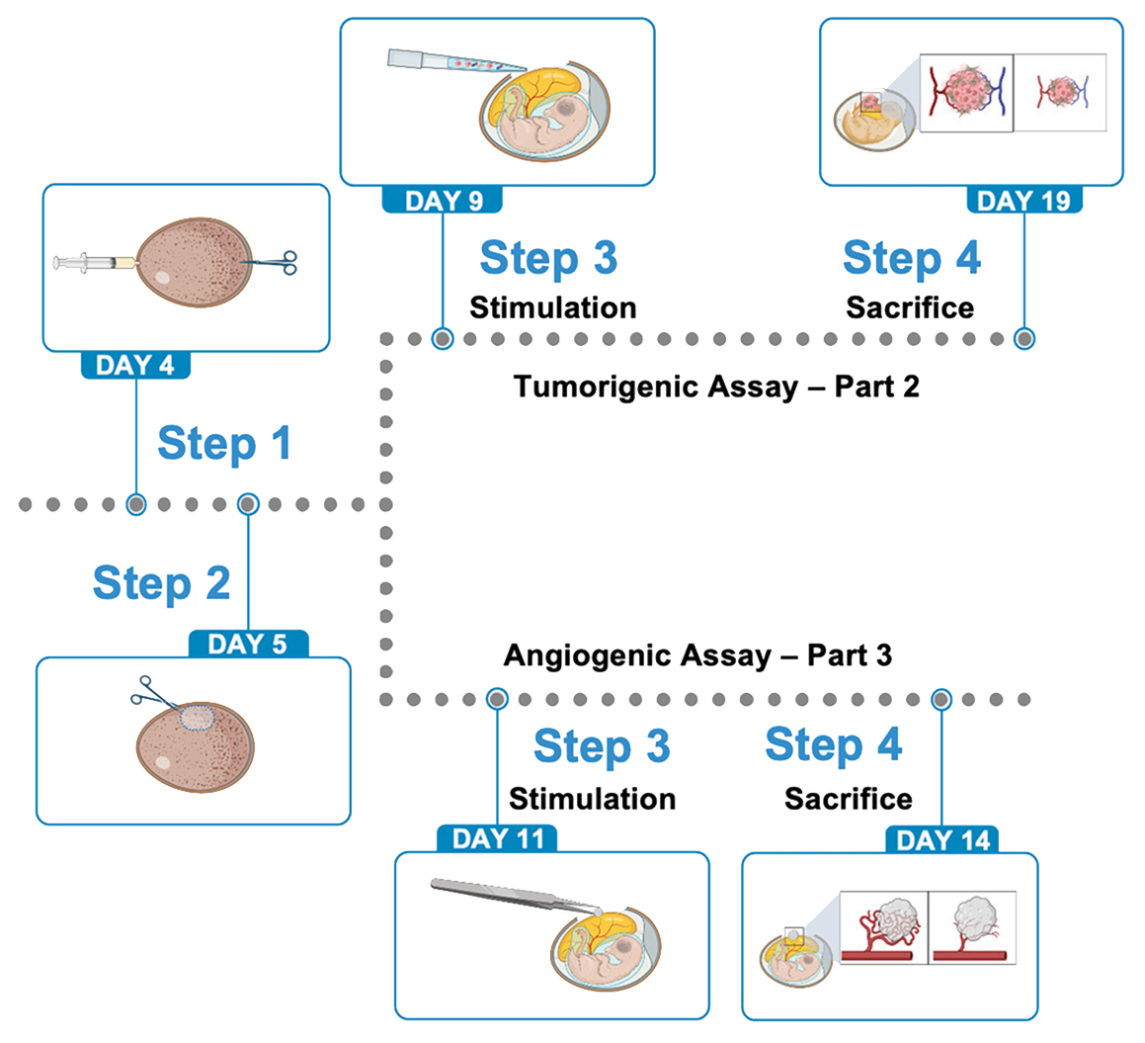
The CAM model for studying tumor growth and angiogenesis involves a structured experimental procedure, typically spanning several days of the chick embryo’s development. The key steps are as follows. Egg preparation and incubation (EDs 1–4) Fertilized chicken eggs are incubated under controlled temperature and humidity, often in a rotating incubator, to allow initial embryonic development. Albumen aspiration and fenestration (EDs 4–5): A small hole is made at the blunt end of the egg (over the air sac), and some albumen is removed using a syringe to lower the CAM and detach it from the inner shell membrane. A window is then cut into the eggshell over a highly vascularized area to expose the CAM beneath. The window is sealed with tape. Tumor cell/stimulus engraftment (EDs 7–9) The tumor cells or bioptic specimens are carefully placed onto the exposed, highly vascularized CAM. The egg is then re-sealed and incubated further without rotation. Tumor growth and angiogenesis development (several days post-engraftment): The tumor grows over several days, developing its own blood supply by inducing angiogenesis from the surrounding CAM vasculature. The development can be monitored daily through the window (reproduced from 11). CAM, chorioallantoic membrane; EDs, embryonic days.

Grafting tumors onto the CAM allows us to study the morphological aspects of the interactions of the tumors with the blood vessels of the host and to examine the identity of the vessels that supply the grafts (**Figure 1**). The formation of peripheral anastomoses between host and preexisting donor vessels is the main and most common mechanism involved in the revascularization of the graft of an embryonic organ onto the CAM, whereas sprouting of CAM-derived vessels into the transplants occurs in the grafts of tumor tissue (12, 13).

The CAM model has a broad range of applications in various cancer types, such as colorectal, prostate, brain, gastric, ovarian, liver, head and neck, and breast cancers, and as a result, it is one of the most used environments for tumor biology and angiogenesis studies (14). Lung cancer was selected as the type of cancer to apply the CAM model because lung cancer has a great prevalence and clinical significance, and lung tumors have a strong angiogenic phenotype that makes the CAM model especially appropriate in investigating tumor–host interactions and anti-angiogenic therapies.​ Moreover, tumor cells can be identified in the CAM, as well as in the internal organs of the embryo, such as the lungs, liver, and brain (15). Moreover, we decided to investigate the CAM application in the study of human lung cancer because, at present, there are no literature data summarizing this topic. General inclusion criteria are limited to peer-reviewed journal articles published in English and within a certain time frame, the last 16 years. Exclusion criteria include studies that do not primarily focus on the CAM model or studies that do not provide enough details on the methodology or have incomplete results necessary for analysis. The entire selection process has been documented using a flowchart, such as the one recommended by the PRISMA statement.

## Anti-angiogenesis in lung cancer

Anti-angiogenesis in lung cancer targets new blood vessel formation that tumors need to grow, using drugs like bevacizumab (Avastin) and ramucirumab (Cyramza) that block vascular endothelial growth factor (VEGF)/VEGF receptor (VEGFR) pathways, improving survival when combined with chemotherapy, especially in non-small cell lung cancer (NSCLC). Two anti-angiogenic agents are currently approved in the United States and/or the European Union (EU) for the treatment of NSCLC following chemotherapy: nintedanib (approved in the EU) [an oral triple angiokinase inhibitor that targets VEGFRs 1–3, platelet-derived growth factor receptors (PDGFRs) α/β, and fibroblast growth factor receptors (FGFRs) 1–3 (16, 17)] and ramucirumab, a human monoclonal antibody that binds to VEGFR2, inhibiting VEGF-induced angiogenesis (18).

Researchers are still exploring optimal use, resistance, toxicity, and combinations with targeted therapies and immunotherapies for better outcomes. In this context, combinations with epidermal growth factor receptor (EGFR) inhibitors or immunotherapy overcome these limitations (19).

Immunotherapy and anti-angiogenic drugs work together to fight lung cancer by disrupting the tumor’s blood supply, while simultaneously unleashing the immune system, creating a powerful combo that shows promising results, especially in advanced NSCLC. Anti-angiogenic drugs, like VEGF inhibitors, starve tumors and reduce the immunosuppressive environment, making it easier for immunotherapy (like PD-1 inhibitors) to activate T cells and attack the cancer, often leading to better survival rates than either treatment alone. This synergy is now a standard approach, with combinations like atezolizumab, bevacizumab, and chemotherapy approved by the Food and Drug Administration (FDA) for first-line treatment (20–22).

## The CAM model in the study of lung cancer tumor growth and angiogenesis

The CAM model provides robust and rapid mechanistic evidence for tumor biology and drug response studies using lung tumor xenografts, using methods comparable to those used for human samples, including histology and immunohistochemistry, molecular analysis, and imaging techniques. The CAM model used in pre-clinical potential research may inform or provide background for later clinical trials and justify the transition to human trials by demonstrating initial efficacy, safety, or mechanistic insights, such as anti-angiogenic drugs.

Miura et al. (23) transplanted lung cancer cell lines and cell line-derived organoids onto the CAM and demonstrated an angiogenic effect, and bevacizumab treatment reduced the number of newly formed blood vessels. Rousset et al. (24) evaluated here whether the isolation of fresh cancer tumor cells (CTCs) from patients with metastatic cancers provides a reliable tumor model after a CAM xenograft. They enrolled patients with lung metastatic cancers. After 48–72 h of culture, the CTCs were engrafted onto the CAM of embryonated chicken eggs at ED 9. The tumors were resected 9 days after engraftment, and histopathological, immunochemical, and genomic analyses were performed. In the lung cancer group, adenocarcinoma was the most frequent histological subtype. Fourteen were positive for PD-L1. It is important to note that the primary difficulties and inconsistencies in making and using CTC xenografts stem from the loss of tumor heterogeneity, the replacement of human stroma with other components, the lack of a functional human immune system in the models, and issues with experimental reproducibility and contamination.

*In vivo* angiogenic response surrounding the SCLC transplantation tumors in CAM was promoted after exogenous HIF-1α transduction. HIF-1α upregulated the expression of angiogenic genes VEGF-A, TNFAIP6, PDGFC, FN1, MMP28, and MMP14 and glycolytic genes GLUT1 and GLUT2. The expression of these angiogenic factors was also upregulated by HIF-1α in the transplantation tumors in CAM, as RT-PCR and Western blotting analysis indicated (25).

Waschkies et al. (26) compared MC-38 colon and A549 lung adenocarcinoma cell grafts grown on the CAM, using quantitative MRI readouts as imaging markers. Different grafts based on the A549 lung adenocarcinoma cell line display distinct phenotypes that can be distinguished and characterized non-invasively *in ovo* using MRI in the living chicken embryo.

Three NSCLC cell lines were used to analyze the anti-angiogenic effect when exposed to metformin alone, pemetrexed alone, or their combination in the CAM model (27). Sodium valproate, a histone deacetylase inhibitor, produces dose-dependent anti-angiogenic and antimigratory effects on lung tumors grafted on the CAM (28).

Li et al. (29) tested human NSCLC xenograft tumors on the CAM. The NSCLC cell lines tested formed a solid tumor. When chemotherapeutic agents and recombinant viruses were tested, the simple application of these agents on the CAM resulted in efficient systemic delivery, and systemic treatment with a combination of pemetrexed and cisplatin inhibited tumor growth (29). The human NSCLC HCC827 cells were engrafted onto the CAM and treated with osimertinib, a third-generation EGFR tyrosine kinase inhibitor, for 7 days (30). They found that tumor growth inversely correlated with osimertinib dosage, and transcriptomic analysis revealed that osimertinib reduced EGFR pathway activity and dampened chemotaxis, immune recruitment, and angiogenesis. MiR-146a-5p-overexpressing NSCLC cells were transplanted onto the CAM, and the xenograft tumor size and angiogenesis of the miR-146a-5p-overexpressing group were lower when compared with the control group (31).

The effect of miR-542-5p on the tumorigenesis of NSCLC was verified in the CAM model. Both tumor growth and angiogenesis were significantly suppressed by miR-542-5p mimic in the CAM (32). When cells derived from the Lewis lung were inoculated onto the CAM at EDs 9–11, large tumors were produced. Although most of these tumors contained mouse cells, they could no longer be transplanted either in C57BL mice or on the CAM. Cloned cells obtained from Lewis lung carcinoma grown *in vitro* produced CAM tumors, which were different from those produced by the parental cells in that the former retained some tumorigenic potential in mice. The phenotype associated with the cloned cell populations was stable *in vitro* and *in vivo* (33).

Human lung cancer cells transfected with pigment epithelium-derived factor (PEDF) were inoculated in the CAM. The tumor volume and the vascular density of the experimental group were significantly smaller than those of the control group (34).

## Other pathological conditions

The CAM model allows scientists to evaluate the effects of potential drugs on lung disease, such as the antifibrotic drugs being tested for pulmonary fibrosis. Perrault et al. (35) implanted xenografts derived from idiopathic pulmonary fibrosis (IPF) on the CAM and evaluated the efficacy of antifibrotic drugs. They demonstrated that the daily treatment of the xenografts with nintedanib and PRI-4050 significantly reduced their size, fibrosis-associated gene expression, and collagen deposition.

## Concluding remarks

The main advantages of the CAM model are the low cost, simplicity, reproducibility, and reliability. An ethical advantage of the CAM model is that the chick embryo is not considered a living animal until ED 17, which can be used without any ethical restriction, and does not require protocol approval by an animal welfare or ethics committee (36).

**Table 1** provides a comparison of the advantages and limitations of the CAM and the mouse xenograft models. Moreover, in contrast to standard mouse models, most cancer cells arrested in the CAM microcirculation survive without cell damage, and many of them complete extravasation within 24 h after injections (37). On the contrary, when standard mouse models of experimental metastasis are used, most of the intravenously injected cancer cells perish in the microcirculation before extravasation (38).

**Table 1:** A comparison of advantages and limitations between the CAM and the mouse xenograft models.

Issue	Mouse xenografts	CAM assay	Advantages CAM	CAM limitations
Duration	4–9 (−12) weeks	3–5, max. 7 days	High throughput	Limited time frame for tumor growth and effects
Costs	High expenses for breeding, keeping, and feeding	Low expenses for eggs and transport	Cost-saving	
Space requirements	High	Low	Space-saving	
Permission requirements	Protocol approval by animal welfare and ethics committee	No approval by welfare or ethics committee required	No administrative burden	

CAM, chorioallantoic membrane.

The limitations of the use of the CAM model are the disparity in immune response in relation to mammalian systems and the applicability of results to human patients. The chick embryo’s immune system remains immature and is naturally immunodeficient during the experimental window. This lack of a fully developed adaptive immune system means that the model cannot accurately replicate the complex immune interactions and responses observed in adult mammalian systems or human patients, which is a major drawback for immunotherapy-related studies. In this context, findings from the CAM model are generally not directly applicable to human clinical practice without further validation in mammalian and clinical models. The model is useful as a bridge between *in vitro* and mammalian *in vivo* studies, but not a total replacement.

The CAM has long been a favored system for the study of tumor biology and tumor angiogenesis because, at the stage of development when generally tumor grafts are placed (6–10 days of incubation), a chick’s immunocompetent system is not fully developed, and the conditions of rejection have not yet been established. All studies for mammalian neoplasms, including lung cancer, have used tumor cell lines, tumor bioptic specimens, cell suspensions derived from tumors, and mouse tumor xenograft bioptic specimens. Compared with mammalian models, where tumor growth takes 3–6 weeks, the chick embryo grows faster. Between 2 and 5 days after tumor implants, the tumor grafts are visible and are supplied with vessels of CAM origin. Tumors grafted onto the CAM remain non-vascularized for a few days, after which they can be penetrated by new blood vessels and begin a phase of rapid growth.

CAM can also be used to study the effects of anti-angiogenic molecules and provides a model to study metastasis. In fact, the chick embryo provides a model to study either spontaneous or experimental metastasis in a considerably shorter time, 7–9 days, compared with 4–10 weeks for most typical murine models (10).

In the meantime, the short duration of the CAM model (7–9 days) is a limiting factor in analyzing the complexity of the human tumor microenvironment and in distinguishing tumor-induced angiogenesis from normal embryonic vascularization. While the CAM model is a powerful tool for studying cancer biology, its ability to fully replicate the tumor microenvironment is constrained by the difficulty of determining if tumor-induced inflammation in the chick parallels the chronic inflammation seen in human cancer. Moreover, human tumor-associated stroma is replaced by chick-derived stroma, altering the tumor’s behavior.

In the case of human glioblastoma multiforme experimental model studies in the CAM, Hagerdon et al. (39) demonstrated that glioblastoma cells formed avascular tumors within 2 days, which progressed through VEGFR2-dependent angiogenesis. Blocking the VEGFR-2 and PDGFR signaling pathways with small-molecule receptor tyrosine kinase inhibitors blocked tumor growth. Moreover, gene regulation analysis during the angiogenic switch by oligonucleotide micro-arrays identified genes associated with tumor vascularization and growth.
